# Burden of clinical syndromes associated with pneumococcal disease in Mexico: a retrospective analysis for 2019

**DOI:** 10.1186/s12879-026-13297-4

**Published:** 2026-04-17

**Authors:** Juan Urrego-Reyes, Patricia Cornejo-Juárez, Jessica Lynn Webster, Paula Pungartnik, Cassia Veiga, Thais das Neves Fraga Moreira, Angélica Carreira dos Santos, Cintia Irene Parellada

**Affiliations:** 1 Outcomes Research Regional Latin America, MSD Colombia, Calle 127A #53A-45 T3 P8, Bogotá, 110111 Colombia; 2 Instituto Nacional de Cancerología, Ciudad de Mexico, Mexico; 3 Outcomes Research Regional Latin America, Merck & Co., Inc, Rahway, NJ USA; 4 Outcomes Research Regional Latin America, MSD Brazil, São Paulo, Brazil; 5 Global Medical & Scientific Affairs (GMSA), MSD Brazil, São Paulo, Brazil; 6 IQVIA Real-World Insights, São Paulo, Brazil

**Keywords:** Pneumococcal disease, Burden, Mortality, Case fatality rate, Hospitalization, Mexico

## Abstract

**Background:**

Pneumococcal disease (PD) represents a significant public health concern, impacting healthcare systems across Latin America. Mexico’s National Immunization Program introduced infant vaccination with the 7‑valent pneumococcal conjugate vaccine (PCV7) in 2008, switching to the 13‑valent pneumococcal conjugate vaccine (PCV13) in 2011. Adult vaccination has been included since 2004. Data on Mexico’s disease burden remain limited; therefore, this study aimed to estimate the burden of clinical syndromes associated with pneumococcal disease in 2019.

**Methods:**

We conducted a retrospective analysis using Mexican administrative hospitalization and mortality databases. Clinical syndromes—including pneumonia, bacteremia, meningitis, and other invasive diseases—were identified using International Classification of Diseases, Tenth Edition (ICD-10) codes. For each syndrome, hospitalization rates, case fatality rates (CFR), and mortality rates were calculated by age group (< 1, 1–4, 5–17, 18–49, 50–59, and ≥ 60 years).

**Results:**

In 2019, 119,628 hospitalizations related to these syndromes were reported, with pneumonia accounting for 78% of cases. Hospitalization rates were highest among infants aged < 1 year, at 557 per 100,000. CFRs increased with age, reaching 27.8% for pneumonia and 70.9% for bacteremia in adults aged ≥ 60 years. Mortality rates were highest for pneumonia in older adults (156.3 per 100,000) and infants aged < 1 year (45.2 per 100,000).

**Conclusions:**

These findings highlight the persistent burden of clinical syndromes associated with PD in Mexico despite vaccination efforts, emphasizing the need for improved immunization strategies, particularly among older adults, and strengthened surveillance to inform public health policies.

**Clinical trial number:**

Not applicable.

**Supplementary Information:**

The online version contains supplementary material available at 10.1186/s12879-026-13297-4.

## Background

Pneumococcal disease (PD) poses a significant public health challenge, disproportionately affecting vulnerable populations such as young children, older adults, and individuals with certain underlying health conditions [1–3]. Despite the availability of effective vaccines, PD continues to be a major cause of preventable illness and death globally. In 2019, it accounted for an estimated 829,000 deaths and over 40 million years of life lost, making it one of the primary contributors to bacterial mortality worldwide [4]. In the same year, in Latin America, more than 84,000 deaths were directly attributable to antimicrobial resistance (AMR) associated with *Streptococcus pneumoniae*. In Mexico specifically, PD was responsible for 8,417 deaths, corresponding to a mortality rate of 7.6 per 100,000 individuals, ranking this pathogen as the fifth most lethal in the country [5]. 

In response, Mexico has progressively expanded its pneumococcal vaccination efforts. The 7-valent pneumococcal conjugate vaccine (PCV7) was introduced into its National Immunization Program (NIP) in 2008 for infants < 1 year (2 + 1 schedule), followed by a switch to PCV13 (also 2 + 1 schedule) in 2011 [6–8]. For adults aged ≥ 60 years, a single dose of the 23-valent pneumococcal polysaccharide vaccine (PPSV23) has been recommended since 2004, with eligibility extended to individuals aged ≥ 2 years with risk conditions [9, 10]. In 2022, NIP guidelines were updated to include PCV13 for healthy older adults and a sequential PCV13 + PPSV23 schedule for individuals with risk conditions [9–12]. Starting in 2025, adults aged ≥ 60 years and those aged 9–59 years with underlying conditions may receive either a single dose of PCV20 or a sequential PCV13 + PPSV23 schedule [13]. 

Vaccination coverage rates (VCR) have followed markedly differently trajectories in pediatric and adult populations. Among children, coverage rose sharply after program implementation—reaching 14% in 2008 and 63% in 2009—and remained consistently above 80% from 2010 onward, before declining slightly to 77% in 2023. In contrast, uptake among older adults has been persistently lower and more erratic, with cross-sectional estimates showing a steady decrease from 44.3% in 2008 to 24.4% in 2022 [14–17]. These trends highlight a sustained high uptake in the pediatric program and persistent gaps in adult pneumococcal vaccination in Mexico.

Assessing the impact of these vaccination efforts depends on the quality and completeness of national disease surveillance data. In Mexico, pneumococcal surveillance is conducted by the Interinstitutional Group for the Surveillance of Vaccine-Preventable Bacterial Diseases (Grupo Interinstitucional para la Vigilancia de Enfermedades Bacterianas Prevenibles por Vacunación - GIVEBPVac), a laboratory-based system that offers valuable insights into serotype distribution and antimicrobial resistance patterns [18]. However, its limited geographical representativeness and reduced number of samples restrict its ability to capture the full spectrum of PD presentations.

To address these limitations, particularly in settings with limited microbiological confirmation, administrative databases based on the International Classification of Diseases (ICD) system can be leveraged. These databases reflect routine clinical practice but often lack pathogen-specific coding. For instance, although *S. pneumoniae* is a leading cause of pneumonia, fewer than 1% of cases are coded as pneumococcal pneumonia; most are recorded under nonspecific codes [19]. Similar issues affect bacteremia and meningitis, resulting in a significant underestimation of the true burden of PD. A syndromic approach—incorporating broader diagnostic codes—has therefore proven valuable and has been employed in other Latin American studies as well as in the Global Burden of Disease (GBD) framework.

Given the significant burden and the limited number of studies quantifying its impact in Mexico, this study aimed to estimate the burden of clinical syndromes associated with PD in Mexico using national-level administrative data from 2019. The findings are intended to inform evidence-based decision-making for immunization policies and public health planning.

## Materials and methods

### Study design and data sources

This was an observational, descriptive, and retrospective study that analyzed national administrative health databases for the year 2019. To assess the burden of clinical syndromes associated with PD, hospitalizations were extracted from the Sectoral database considering the main cause. This database includes records from major Mexican social security and federal health providers—Instituto Mexicano del Seguro Social (IMSS), Instituto de Seguridad y Servicios Sociales para los Trabajadores del Estado (ISSSTE), Instituto de Salud para el Bienestar (Bienestar), Secretaría de la Defensa Nacional (DEFENSA), Secretaría de Marina (MARINA), and Petróleos Mexicanos (Pemex) —which together constitute the main public social security institutions, covering almost the entire insured population and approximately 70% of the Mexican population (about 90 million people) [20, 21]. The database captures discharges across all three levels of care—basic preventive, specialized, and high complexity—nationwide but does not include data from the private sector, which represents around 2% of the population. Information on outpatient visits was not available in the datasets used. Mortality data, based on the underlying cause of death, were sourced from the National Mortality Epidemiological and Statistical System (Subsistema Epidemiológico y Estadístico de Defunciones - SEED), which compiles information from all death certificates nationwide. Population estimates for 2019 were sourced from the National Population Council of Mexico (Consejo Nacional de Población - CONAPO).

The Mexican Ministry of Health compiles all databases using standardized procedures for data collection and validation, which include ICD-10 coding and internal consistency checks. The data utilized in this study were anonymized and aggregated. The protocol was approved by the Instituto Nacional de Cancerología (INCAN) ethics committee (INCAN/CEI/690/21 on July 8, 2021), in accordance with local regulations. It was also registered with COFEPRIS under number 12 CEI 0901411 and CONBIOETICA-09-CEI-002-20160413.

### Study population and case definitions

Severe clinical syndromes associated with PD were identified using ICD-10 for pneumonia (J13-J18), bacteremia (A40-A41), meningitis (G00.1-G00.9), and other invasive diseases (J86-J91) (Supplementary Table S1). Consistent with previous evidence from Latin America showing that excluding pneumonia of known viral origin (J12) provides more specific estimates of pneumococcal vaccine impact, we restricted our pneumonia definition to ICD-10 codes J13–J18 to better capture syndromes more likely attributable to *S. pneumoniae* [22]. The study population included individuals of all ages and was stratified into the following age groups: <1 year, 1–4 years, 5–17 years, 18–49 years, 50–59 years, and ≥ 60 years. The < 1 year category included all infants under 12 months, including neonates. For hospitalization data, the primary cause ICD-10 diagnosis was employed, while for mortality, the underlying cause of death was considered. The year 2019 was selected to avoid confounding effects from the COVID-19 pandemic and to ensure data completeness.

### Statistical analysis

The primary outcomes assessed were the hospitalization rate, the in-hospital case fatality rates (CFR), and the mortality rate. Absolute numbers and proportions of hospitalizations, in-hospital deaths, and overall fatalities were calculated for each clinical syndrome associated with PD. Annual hospitalization and mortality rates were estimated using the number of inpatient cases and deaths as numerators and population estimates as denominators. All rates were reported per 100,000 population. Cases missing age data were included in overall total counts but excluded from age-specific rate calculations. The number of cases without age information for each syndrome was reported in the corresponding table. In-hospital CFRs were computed by dividing the number of inpatient deaths by the total number of hospitalizations for each clinical syndrome, with results expressed as percentages. The 95% confidence intervals (CIs) were determined using the Poisson distribution. All statistical analyses were conducted using Python version 3.10.12 (Python Software Foundation).

## Results

### Annual hospitalization rate

In 2019, a total of 5,774,867 all-cause hospitalizations were recorded in the Sectoral database across all age groups in Mexico. Clinical syndromes associated with PD accounted for 119,628 of these admissions, corresponding to 2.1% of overall hospitalizations. Among adults aged ≥ 60 years, these syndromes comprised 3.8% of hospitalizations, while in children aged < 5 years they represented 8.3%. Of the PD-associated hospitalizations, 93,149 (77.8%) cases were attributed to pneumonia, while 26,479 (22.1%) cases were due to other severe presentations, which included 14,288 cases of bacteremia, 1,258 cases of meningitis, and 10,933 cases of other invasive diseases (Table 1).

Hospitalizations were most prevalent at the extremes of age: children aged < 5 years accounted for 32.1% of admissions, while adults aged ≥ 60 years represented 38.0% (Table 1). Pneumonia was the leading clinical syndrome across all age groups. Among children, infants aged < 1 year exhibited the highest hospitalization rates per 100,000, with 557.9 for pneumonia and 25.4 for bacteremia. Children aged 1–4 years had the next highest rate of pneumonia-related hospitalizations at 286.9 per 100,000. The lowest hospitalization rates for all syndromes were observed in individuals aged 5–17 years. Adults aged ≥ 60 years had the highest hospitalization rates across all syndromes: 226.2 per 100,000 for pneumonia, 61.2 for bacteremia, and 37.1 for other invasive diseases.

**Table 1:** Number of hospitalizations and hospitalization rates per 100,000 for clinical syndromes associated with PD by age group in Mexico, 2019

Age group (years)	Pneumonia		Bacteremia		Meningitis		Other invasive disease*	
	Cases	Rate (95% CI)	Cases	Rate (95% CI)	Cases	Rate (95% CI)	Cases	Rate (95% CI)
All ages	93,149	73.6	14,288	11.3	1,258	1.0	10,933	8.6
		(73.5–73.7)		(11.2–11.3)		(0.9–1.1)		(8.6–8.7)
< 1	12,011	557.9	546	25.4	136	6.3	17	0.8
		(557.5–558.4)		(24.9–25.8)		(5.9–6.7)		(0.4–1.2)
1–4	25,075	286.9	363	4.2	105	1.2	98	1.1
		(286.7–287.1)		(3.9–4.4)		(1.0–1.4)		(0.9–1.3)
5–17	6,792	23.5	385	1.3	169	0.6	203	0.7
		(23.4–23.6)		(1.2–1.5)		(0.5–0.7)		(0.6–0.8)
18–49	10,977	18.2	2,498	4.1	482	0.8	3,204	5.3
		(18.1–18.2)		(4.1–4.2)		(0.7–0.9)		(5.2–5.4)
50–59	6,778	54.7	1,965	15.8	174	1.4	2,247	18.1
		(54.5–54.9)		(15.7–16.0)		(1.2–1.6)		(17.9–18.3)
≥ 60	31,516	226.2	8,531	61.2	192	1.4	5,164	37.1
		(226.0–226.3)		(61.1–61.4)		(1.2–1.5)		(36.9–37.2)

CI: confidence interval

*Includes pneumococcal arthritis, pyothorax / empyema, pleural effusion and S. pneumoniae classified elsewhere (see Supplementary Table S1)

### In-hospital case fatality rates

**Fig. 1:**
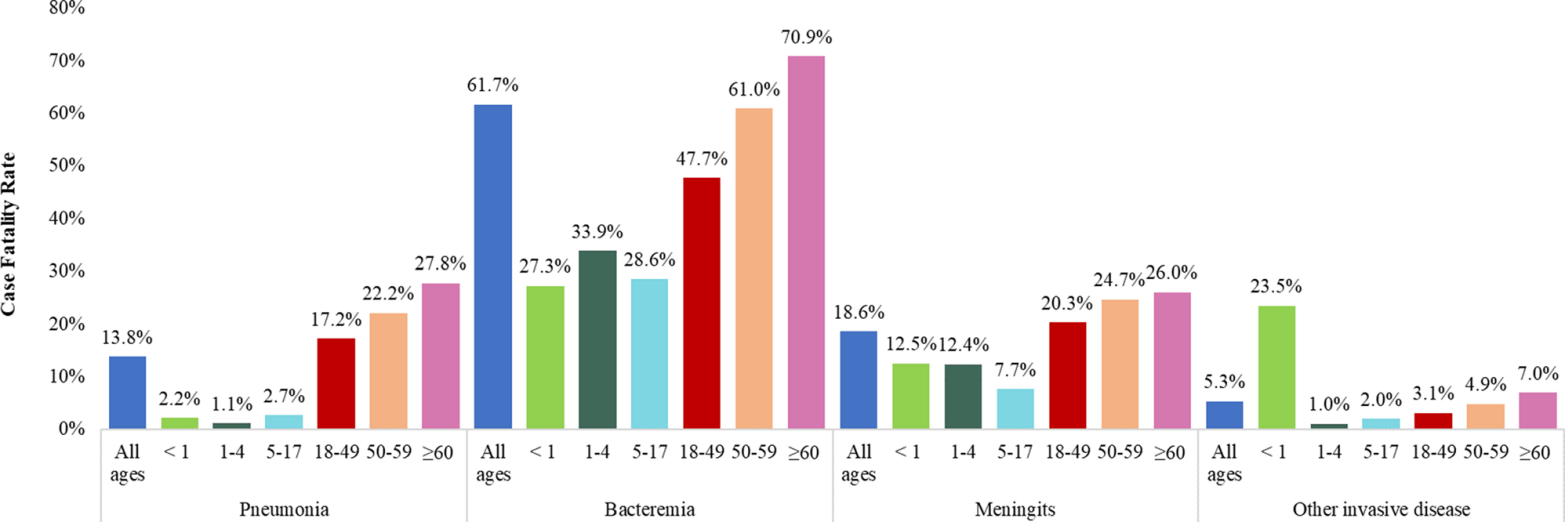
In-hospital case fatality rates for clinical syndromes associated with PD by age group in Mexico, 2019

In-hospital CFRs for pneumonia increased progressively with age. Among individuals aged < 18 years, the CFR was below 3.0%. This rate rose to 17.2% in the 18–49 age group and reached 27.8% in adults aged ≥ 60 years (Fig. 1). For bacteremia, the overall in-hospital CFR was 61.7%. The highest CFR was observed in adults aged ≥ 60 years at 70.9%, followed by 61.0% in those aged 50–59 years, and 47.7% in individuals aged 18–49 years. For meningitis, the highest CFRs were observed among individuals aged ≥ 18 years, reaching up to 26.0% in adults aged ≥ 60 years (Fig. 1).

### Mortality rates

In 2019, a total of 36,937 deaths were associated with clinical syndromes commonly caused by PD in Mexico. Pneumonia accounted for most of these deaths (29,924; 81.0%), followed by bacteremia (5,818; 15.8%), other invasive diseases (773; 2.1%), and meningitis (422; 1.1%) (Table 2). Of these deaths, 5.5% occurred in children aged < 5 years, 23.3% in individuals aged 18–59 years, and 70.9% in adults aged ≥ 60 years.

The overall mortality rate for pneumonia was 23.6 per 100,000 inhabitants, while rates for bacteremia, meningitis, and other invasive diseases were 4.6, 0.3, and 0.6 per 100,000, respectively. The highest mortality burden was observed in adults aged ≥ 60 years, with pneumonia-related deaths reaching 156.3 per 100,000, and bacteremia-related deaths 26.1 per 100,000. Infants aged < 1 year also experienced elevated mortality rates, particularly for pneumonia (45.2 per 100,000) and bacteremia (18.7 per 100,000). In contrast, mortality rates were lowest among children aged 5–17 years, across all syndromes.

**Table 2:** Numbers of deaths and mortality rates per 100,000 inhabitants for clinical syndromes associated with PD by age group in Mexico, 2019

Age group (years)	Pneumonia		Bacteremia		Meningitis		Other invasive disease	
	Deaths	Rate (95% CI)	Deaths	Rate (95% CI)	Deaths	Rate (95% CI)	Deaths	Rate (95% CI)
All ages*	29,924	23.6 (23.6–23.7)	5,818	4.6 (4.5–4.6)	422	0.3 (0.3–0.4)	773	0.6 (0.6–0.7)
< 1	972	45.2 (44.7–45.6)	402	18.7 (18.2–19.1)	32	1.5 (1.1–1.9)	6	0.3 (0.0–0.7)
1–4	504	5.8 (5.6–6.0)	106	1.2 (1.0–1.4)	21	0.2 (0.0–0.4)	5	0.1 (0.0–0.3)
5–17	226	0.8 (0.7–0.9)	111	0.4 (0.3–0.5)	25	0.1 (0.0–0.2)	5	0.0 (0.0–0.1)
18–49	3,445	5.7 (5.6–5.8)	814	1.3 (1.3–1.4)	148	0.2 (0.2–0.3)	136	0.2 (0.1–0.3)
50–59	2,915	23.5 (23.3–23.7)	714	5.8 (5.6–6.0)	70	0.6 (0.4–0.7)	133	1.1 (0.9–1.2)
≥ 60	21,775	156.3 (156.1–155.4)	3,640	26.1 (26.0–26.3)	121	0.9 (0.7–1.0)	486	3.5 (3.3–3.6)

*125 deaths were missing ages: 87 for pneumonia, 31 for bacteremia, 5 for meningitis, and 2 for other invasive disease

CI: confidence interval

## Discussion

In this national retrospective analysis of inpatient and mortality databases for 2019, we found that clinical syndromes associated with PD imposed a substantial burden in Mexico. That year, there were 119,628 hospitalizations and 36,937 deaths associated with clinical syndromes commonly caused by *S. pneumoniae*. Pneumonia accounted for the majority of hospitalizations (77.8%) and deaths (81.0%), with CFR rising from < 3% in children to 27.8% in adults ≥ 60 years. Bacteremia had an overall CFR of 61.7%, peaking at 70.9% in adults ≥ 60 years. These findings highlight the continued burden of PD in both pediatric and older adult populations, despite existing vaccination efforts.

Multiple studies have confirmed the substantial direct impact of pediatric PCV programs on reducing pneumococcal disease burden in children under five years across Latin America [7, 23, 24]. Oliveira et al., reported significant declines in pneumonia mortality among children aged 2–59 months following PCV introduction in most of the ten countries analyzed, including Mexico [23]. However, these benefits were not mirrored in older populations [24]. Indeed, a time-series analysis showed that, between 2016 and 2019, pneumonia mortality among adults ≥ 60 years increased by 13.1% annually.^24^ These findings are consistent with multicountry analyses, which reported limited herd effects and rising mortality in older adults across Latin America [19, 25, 26]. The persistence of non-vaccine serotypes and low adult vaccination coverage (< 25%) likely contribute to this gap.

Mexican surveillance data from 2012 to 2023 indicate that most PCV13 vaccine serotypes have been eliminated from circulation in children, with the exception of serotypes 3, 19 A, and 19 F, which continue to cause around one-third of invasive and non-invasive disease cases [7, 18]. According to the 2023 GIVEBPVac report, residual disease caused by these serotypes remains substantial among children aged < 5 years, with clear serotype 19 A predominance [18]. Serotype 3 shows an age-related increase, becoming the most prevalent among adults aged ≥ 60 years.

In response to this evolving epidemiological landscape, Mexico’s adult vaccination guidelines have undergone significant changes. From a single-dose PPSV23 recommendation for all adults ≥ 60 years (2004–2018), the policy evolved to include risk-based schedules and the introduction of PCV13 between 2022 and 2024 [9, 10]. The most recent 2025 guidelines propose PCV20 as an alternative to the PCV13/PPSV23 combination for adults aged 19–59 years at risk, reflecting a shift toward broader serotype coverage and simplified schedules [9, 10]. Notably, approximately 50% of PD cases in older adults are caused by serotypes covered by PCV20, and over 70% by PCV21, a pneumococcal vaccine designed for adults that has eight unique serotypes not included in PCV20 [18]. 

Alongside immunization and tracking serotype distribution, AMR is an increasingly important driver of pneumococcal disease burden and clinical outcomes. *S. pneumoniae* remains one of the leading pathogens associated with AMR-attributable deaths, particularly in low- and middle-income countries [4]. Mexico has long reported high penicillin resistance in *S. pneumoniae*, and recent data show emerging resistance to trimethoprim-sulfamethoxazole and meropenem [27]. Global and regional analyses reveal substantial variability in resistance to penicillins, macrolides and third-generation cephalosporins across Latin America, reflecting heterogeneous antibiotic use and stewardship practices [28]. Inappropriate outpatient prescribing for respiratory infections continues to drive selective pressure, reinforcing the bidirectional relationship between antibiotic use and resistance [29]. Consequently, integrating AMR surveillance with vaccination strategies is essential: higher-valency PCVs can reduce disease due to serotypes historically associated with multidrug resistance, while robust antimicrobial stewardship is needed to preserve treatment effectiveness and maximize the overall benefit of vaccination [30, 31]. 

From a strategic public policy perspective, the insights generated by this study warrant particular attention to the population of older adults (≥ 60 years). Our findings underscore the urgent need to strengthen healthcare infrastructure and expand adult vaccination coverage in this age group, especially considering that rapid population aging in Mexico coexists with persistent structural inequities that disproportionately affect older individuals [32]. PD syndromes—particularly bacteremia and meningitis—are associated with high clinical severity and an increased risk of long-term sequelae [33]. These characteristics emphasize the importance of timely diagnosis, appropriate antimicrobial therapy, and robust preventive strategies focused on older adults, who remain especially vulnerable in settings with limited access to specialized care and diagnostic resources.

Several limitations should be acknowledged when interpreting the findings of this study. Variability in the accuracy and completeness of national database records may have influenced the results. Hospitalization data were derived from major Mexican social security and federal health providers, covering approximately 70% of the population. Therefore, findings primarily reflect this subset and should be interpreted with caution when generalizing to individuals affiliated to other healthcare systems or those without coverage. Furthermore, the analysis relied on administrative databases for a single calendar year, restricting the ability to account for annual fluctuations in disease burden. Factors such as changes in vaccination schedules, VCR, shifts in circulating serotypes, or broader temporal trends could not be assessed, which may affect the generalizability of the findings.

Similarly, although *S. pneumoniae* becomes a more prominent cause of severe disease in older infants and young children, accounting for an estimated attributable fraction of up to 21.6% among those under 5 years, it is essential to interpret pneumonia hospitalization rates in the < 1-year age group with caution [34–37]. This category includes neonates, in whom pneumonia frequently results from mixed viral etiologies such as respiratory syncytial virus, influenza, and enterovirus, as well as bacterial pathogens including group B *Streptococcus*, *Escherichia coli*, and other Gram-negative organisms, thereby reducing the relative likelihood of pneumococcal etiology in this age group [34–37]. 

Future research should aim to include multi-year datasets and, where feasible, incorporate laboratory-confirmed cases and information from both public and private sectors. Such enhancements would improve precision, allow evaluation of temporal dynamic, and provide a more comprehensive assessment of epidemiological patterns and healthcare utilization. The strengths of this study include the use of Mexico’s national death registration data, which are classified as high quality according to World Health Organization (WHO) standards, enhancing the credibility and reliability of our findings [38]. Additionally, this study included the use of a syndromic ICD-10 approach, offering a pragmatic and valuable method for estimating disease burden in low-income and middle-income countries, where underreporting and limited microbiological confirmation are common challenges [19, 23, 25]. 

The GBD 2021 study underscores the central role of *S. pneumoniae*, which accounts for the largest proportion of global lower respiratory infection (LRI) episodes and deaths [39]. In Mexico, it was responsible for roughly 18–25% of LRI episodes and 16–25% of LRI-related deaths across all age ages —likely representing an even greater share in our more specific, non‑viral pneumonia definition [39]. Prospective data from Latin America consistently identified *S. pneumoniae* as the leading cause of all-cause pneumonia and bacterial meningitis in adults, and validation studies show that ICD-10 codes for pneumonia and sepsis capture a substantial proportion of confirmed pneumococcal cases [40–45]. Taken together, these findings reinforce the utility of a syndromic, ICD-10-based approach for public health surveillance, particularly where routine laboratory confirmation is limited [16]. 

## Conclusion

Given the limited research quantifying the impact of PD in Mexico, this study underscores the sustained burden of clinical syndromes associated with PD, particularly at the extremes of age. Adults aged ≥ 60 years exhibited the highest CFR across most presentations, recorded more pneumonia-related deaths than any other age group, and had an age-specific mortality rate of 156.3 per 100,000. The findings provide robust, national evidence to support informed decision-making in immunization policies and broader public health planning. Rather than presenting a conclusive overview of PD in Mexico, this study reinforces the epidemiological basis for public health discussions and encourages continued research and surveillance as pathways toward achieving wider health and socioeconomic goals.

## Supplementary Information

Below is the link to the electronic supplementary material.

Supplementary Material 1

## Data Availability

All data used in this study are publicly available from official Mexican government sources. Hospitalization data were collected from Sectoral database. Mortality data were obtained from the National Mortality Epidemiological and Statistical System (SEED). All databases are available on the General Direction of Health Information (DGIS) website: http://www.dgis.salud.gob.mx/contenidos/basesdedatos/Datos_Abiertos_gobmx.html.

## Abbreviations

CFR: Case fatality rate
CI: Confidence interval
CONAPO: Consejo Nacional de Población
COFEPRIS: Comisión Federal para la Protección contra Riesgos Sanitarios
DEFENSA: Secretaría de la Defensa Nacional
GBD: Global Burden of Disease
GIVEBPVac: Grupo Interinstitucional para la Vigilancia de Enfermedades Bacterianas Prevenibles por Vacunación
ICD-10: International Classification of Diseases, 10th Revision
IMSS: Instituto Mexicano del Seguro Social
INCAN: Instituto Nacional de Cancerología
ISSSTE: Instituto de Seguridad y Servicios Sociales para los Trabajadores del Estado
LRI: Lower respiratory infection
MARINA: Secretaría de Marina
NIP: National Immunization Program
PCV: Pneumococcal conjugate vaccine
PCV7: 7-valent pneumococcal conjugate vaccine
PCV13: 13-valent pneumococcal conjugate vaccine
PCV20: 20-valent pneumococcal conjugate vaccine
PCV21: 21-valent pneumococcal conjugate vaccine
PD: Pneumococcal disease
Pemex: Petróleos Mexicanos
PPSV23: 23-valent pneumococcal polysaccharide vaccine
SEED: Subsistema Epidemiológico y Estadístico de Defunciones
*S. pneumoniae*: Streptococcus pneumoniae
VCR: Vaccination coverage rate
WHO: World Health Organization
