# Supplementary material for: Burden of clinical syndromes associated with pneumococcal disease in Mexico: a retrospective analysis for 2019
Source: BMC Infect Dis. 2026 Apr 17;26:1122. doi: 10.1186/s12879-026-13297-4 (PMC13261829; doi:10.1186/s12879-026-13297-4)
Supplement: Supplementary file 1 — Supplementary Material 1 [file 12879_2026_13297_MOESM1_ESM.docx]

**Burden of clinical syndromes associated with pneumococcal disease in Mexico: A 2019 analysis**

**Supplementary Material**

Table S1. Specific ICD-10 codes used for each clinical syndrome associated with pneumococcal disease and corresponding hospitalization and mortality distributions in Mexico, 2019.

| **Clinical syndrome** | **Diagnosis** | **ICD-10 code** | **Hospitalizations** | | **Deaths** | |
| --- | --- | --- | --- | --- | --- | --- |
|  |  |  |  |  |  |  |
|  |  |  | **Count** | **Distribution** | **Count** | **Distribution** |
| Pneumonia | Pneumococcal pneumonia | J13 | 380 | 0.4% | 5 | 0.0% |
|  | Bacterial pneumonia, unspecified | J15, J15.0-9 | 12,683 | 13.6% | 2,706 | 9.0% |
|  | Pneumonia due to other infectious organisms, not elsewhere classified | J16, J16.0, J16.8 | 289 | 0.3% | 15 | 0.1% |
|  | Pneumonia in diseases classified elsewhere | J17, J17.0-3, J17.8 | 195 | 0.2% | 0 | 0.0% |
|  | Pneumonia or bronchopneumonia, organism unspecified | J18.0, J18.1, J18.9 | 79,602 | 85.5% | 27,198 | 90.9% |
|  | **Total** | | 93,149 | 100.0% | 29,924 | 100.0% |
| Bacteremia | Streptococcal septicemia, unspecified | A40, A40.0-3, A40.8-9 | 98 | 0.7% | 2 | 0.0% |
|  | Other septicemia | A41, A41.0-5, A41.8-9 | 13,514 | 94.6% | 5,651 | 97.1% |
|  | Other bacterial infection, unspecified location | A49, A49.0-3, A49.8-9 | 676 | 4.7% | 165 | 2.8% |
|  |  | **Total** | 14,288 | 100.0% | 5,818 | 100.0% |
| Meningitis | Pneumococcal meningitis | G00.1 | 9 | 0.7% | 4 | 0.9% |
|  | Streptococcal meningitis | G00.2 | 9 | 0.7% | 3 | 0.7% |
|  | Other bacterial meningitis | G00.8 | 54 | 4.3% | 5 | 1.2% |
|  | Unspecified bacterial meningitis | G00.9 | 547 | 43.5% | 224 | 53.1% |
|  | Meningitis, unspecified | G03.9 | 639 | 50.8% | 186 | 44.1% |
|  |  | **Total** | 1,258 | 100.0% | 422 | 100.0% |
| Other invasive diseases | Pneumococcal arthritis | M00.1 | 2 | 0.0% | 0 | 0.0% |
|  | Pyothorax / empyema | J86, J86.0, J86.9 | 1,332 | 12.2% | 297 | 38.4% |
|  | Pleural effusion | J90, J91 | 9,599 | 87.8% | 476 | 61.6% |
|  | *S. pneumoniae* classified elsewhere | B95.3 | 0 | 0.0% | 0 | 0.0% |
|  |  | **Total** | 10,933 | 100.0% | 773 | 100.0% |
